# Editorial: Abdominal and Perianal Fistulizing Crohn's Disease: Imaging, Surgical Techniques and Basic Research

**DOI:** 10.3389/fsurg.2022.952874

**Published:** 2022-06-28

**Authors:** Gaetano Luglio, Francesca Paola Tropeano, Gianluca Pagano, Michele Cricrì

**Affiliations:** Endoscopic Surgery Unit, Integrated Department of Gastrointestinal Disease, School of Medicine, University of Naples Federico II, Naples, Italy

**Keywords:** IBD – inflammatory bowel disease, fistulizing crohn’s disease, perianal crohn disease, surgery, stem cell, abscess

**Editorial on the Research Topic**

**Abdominal and Perianal Fistulizing Crohn’s Disease: Imaging, Surgical Techniques and Basic Research**

Crohn’s disease (CD) is an idiopathic inflammatory bowel disease characterized by chronic transmural inflammation typically affecting the distal ileum and the proximal colon.

In penetrating CD (PCD), the formation of fistulas derives from the transmural migration of bacteria from the gut lumen to contiguous tissues, giving rise to a variety of clinical conditions, ranging from an abdominal inflammatory mass to enteric fistulas up to perianal fistulas (1–3).

The prevalence of PCD reaches 16% in the adult CD population, and there is a strong association between abdominal and perianal fistulizing disease, especially in Crohn’s colitis (4, 5).

For its complex and multiform nature, PCD represents a challenge among the multidisciplinary team, posing several dilemmas regarding timing and role of medical, radiological, and surgical approaches.

When technically feasible, preoperative optimization of intra-abdominal abscesses should be considered; indeed, operating on a CD complicated by an abscess or a phlegmon without adequate down-staging is difficult and likely to require laparotomy, resection of healthy bowel or organs adherent to the inflammatory mass, and the creation of an ostomy due to the higher risk of anastomotic dehiscence (6–9).

Preoperative optimization aims to reduce the abdominal sepsis and bridge the patient to the surgical intervention in better clinical conditions: antibiotic treatment, oral diet restriction and nutritional support are the cornerstones of medical improvement in the preoperative management of abdominal abscesses smaller than 3 cm (10).

For abscesses larger than 3 cm, percutaneous drainage (PTD) should be considered. Compared to surgical drainage, PTD demonstrated lower complication rates and shorter hospital stay, allowing to perform an elective surgical intervention with lower stoma and morbidity rate.

Although the conservative approach is always more feasible for treating abdominal PCD, it is not clear whether this may be enough or serve as bridge to the definitive surgical intervention.

Indeed, PTD could either resolve the intra-abdominal abscess or convert it into an enterocutaneous fistula, which can be addressed surgically without the need of stoma formation.

However, even after adequate non-surgical optimization, most patients will require surgery to treat their underlying PCD. A waiting period of 6–8 weeks after allows a more profound stabilization of patient’s weight loss, anemia, and hypoalbuminemia. Laparoscopic approach should be preferred, even in more complex cases; moreover, preoperative PTD itself makes laparoscopic surgery more feasible (6). With respect to its application in uncomplicated disease, laparoscopic approach for PCD presents similar outcomes in terms of morbidity and hospital stay; nevertheless, it is associated with longer operative times and higher risk of conversion and diverting stoma.

There are several clinical scenarios in which surgery may be performed for PCD.

In presence of an inflammatory mass, if disease and healthy loops are distinguishable, adequate separation and resection can be accomplished; on the contrary, if it is not possible to separate diseased and non-disease intestinal segments, then the best option may be a diverting stoma, switching off the acute inflammation and allowing a less radical surgery later in time.

In case of intra-abdominal abscess, surgery is indicated if sepsis cannot be controlled non-surgically or after adequate pre-operative optimization, which may permit resection and anastomosis rather than stoma formation, which is instead the rule in the former scenario.

Perianal Fistulizing Crohn’s Disease (PFCD) is a highly disabling disease phenotype, whose occurrence varies between 14% and 23% in patients affected by CD, which encompasses a wide variety of entities, including both fistulising lesions (abscesses, perianal or rectovaginal fistulas) and non-fistulising ones (fissures, deep ulcers, anorectal strictures, skin tags, or haemorrhoids). Moreover, the clinical impact of these entities can also vary significantly from asymptomatic and mild diseases to severe scenarios (11, 12).

Perianal fistulas are usually classified by their relationship to the anal sphincter complex letting the surgeon to opt for the best surgical approach (13). An immediate drainage is the treatment of choice when a perianal abscess is present. Simple asymptomatic fistulas usually do not require further treatment once perianal sepsis is under control (14). In cases of symptomatic fistulas or complex disease, a “cone-like” fistulectomy with a draining seton is usually advised. Hand in hand with surgery, the introduction of biologics has remarkably empowered the management of perianal CD, making a combined surgical and medical approach the standard of care (6, 15, 16). Seton placement enhance fibrosis, preventing premature skin closure and ensuring controlled long-term fistula drainage while maintaining the external anal sphincter's integrity. Patients’ biologic therapy might be administered with a lower risk of septic complications, enhancing the effectiveness of medical therapy. However, there is still some debate concerning the quality of life of patients with a seton placed.

As a matter of fact, perianal fistulas healing is still an unmet need in IBD care, being healing rates about 52% despite all the efforts of combined multidisciplinary treatments. Additional surgical techniques, such as advancement flaps or the LIFT operation, do not always guarantee healing, leaving a considerable number of patients with a draining fistula.

Novel therapeutic tools are arising. Among them, the role of local injection of mesenchymal stem cells (MSCs) is emerging as promising.

Whether autologous or allogeneic MSCs constitute the best cellular product remains unanswered from the clinical trials published to date. Some authors advocate the use of autologous MSCs because of safety concerns. However, there is substantial evidence that not all autologous MSCs are equivalent in terms of proliferative and functional properties. Additionally, it was shown that MSCs harvested from adipose tissue in patients with CD exhibit reduced immunosuppressive capabilities when compared with MSCs from healthy donors. The challenges of autologous MSCs include not only the difficulties and possible complications of harvesting, but also harvesting variability and potential waste of resources.

In this research topic, several aspects of PCD have been explored.

Myrelid et al. provided readers with an overview of penetrating abdominal CD and how this scenario should be managed, while Reylonds et al. discuss about surgical strategies to prevent CD recurrences.

Luceri et al. aimed to correlate the occurrence of intestinal fistulization to the plasma levels of MMP9, and of miR-126.

As for PFCD, Colombo et al. shared their experience with MSCs while quality of life issues related with seton placement are investigated by Angriman et al.
